# Supplementary material for: Clinical Presentation of Left Ventricular Noncompaction Cardiomyopathy and Bradycardia in Three Families Carrying HCN4 Pathogenic Variants
Source: Genes (Basel). 2022 Mar 8;13(3):477. doi: 10.3390/genes13030477 (PMC8949387; doi:10.3390/genes13030477)
Supplement: Supplementary file 1 [file genes-13-00477-s001.zip › genes-1622434-supplementary.pdf]

Supplementary Material

Cardiomyopathy-related genes analysed in this study:

A/ Genes included in TruSight One panel: *AARS2, ABCC9, ACAD9, ACADVL, ACTA1, ACTC1, ACTN2, ACVR2B, ADCY6, AGK, AGL, AKAP9, AKT3, ANK2, ANKRD1, ATP5F1E, ATPAF2, BAG3, BMPR2, BOLA3, BRAF, CACNA1C, CACNA2D1, CACNB2, CALM1, CALR3, CASQ2, CAV3, CAVIN4, CBL, CFC1, CHD7, CITED2, COA5, COQ2, COQ8A, COQ9, COX10, COX14, COX15, COX6B1, CPT1A, CPT2, CRELD1, CRYAB, CSRP3, CTF1, CTNNA3, DES, DLD, DMD, DMPK, DNAJC19, DNM1L, DOLK, DPP6, DSC2, DSG2, DSP, DTNA, ELN, ELP1, EMD, ETFB, EYA4, FHL1, FHL2, FKTN, FLNA, FLNC, FOXC1, FOXD4, FOXH1, FOXRED1, FXN, GAA, GATA4, GATA6, GATAD1, GBE1, GDF1, GJA1, GJA5, GLA, GLB1, GNAI2, GPD1L, GUSB, HAND2, HCCS, HCN4, HFE, HRAS, HSPB7, ILK, IRX4, JAG1, JPH2, JUP, KCNA5, KCND3, KCNE1, KCNE2, KCNE3, KCNH2, KCNJ2, KCNJ5, KCNJ8, KCNQ1, KLF5, KRAS, LAMA2, LAMA4, LAMP2, LDB3, LEFTY2, LIAS, LMNA, LRPPRC, MAP2K1, MAP2K2, MED13L, MLYCD, MMUT, MRPL3, MTO1, MYBPC3, MYH6, MYH7, MYL2, MYL3, MYLK2, MYO6, MYOM1, MYOZ2, MYPN, NDUFA10, NEBL, NEXN, NF1, NKX2-5, NKX2-6, NODAL, NOTCH1, NOTCH2, NPPA, NRAS, NSD1, OBSCN, PCCA, PCCB, PDLIM3, PGK1, PIK3CA, PIK3R2, PITX2, PKP2, PLN, POLG, PRKAG2, PSEN1, PSEN2, PTPN11, RAF1, RASA1, RBM10, RBM20, RYR2, SCN10A, SCN1B, SCN2B, SCN3B, SCN4B, SCN5A, SCO2, SDHA, SDHD, SEMA3E, SGCA, SGCB, SGCD, SHOC2, SLC22A5, SLC25A20, SLC25A3, SLC25A4, SMAD2, SMAD6, SNTA1, SOD2, SOS1, SPRED1, SYNE1, SYNE2, TAB2, TAFAZZIN, TBX1, TBX20, TBX3, TBX5, TCAP, TFAP2B, TGFB3, TLL1, TMEM43, TMEM70, TMPO, TNNC1, TNNI3, TNNT2, TPM1, TRDN, TRPM4, TSFM, TTN, TTR, TXNRD2, VCL, XK, YARS2, ZFPM2, ZIC3*

B/ Genes included in CMHI 1000NGS panel: *A2ML1, AARS2, ACAD9, ACADVL, ACTA1, ACTC1, ACTN2, AGK, AGL, ANK2, ATP5F1E, ATPAF2, BAG3, BMPR2, BOLA3, BRAF, CACNA1C, CACNB2, CALR3, CASQ2, CAV3, CBL, CFC1, CHD7, CITED2, COA5, COQ2, COQ8A, COQ9, COX10, COX14, COX15, COX6B1, CPT1A, CPT2, CRELD1, CRYAB, CSRP3, DES, DLD, DMD, DNAJC19, DSC2, DSG2, DSP, DTNA, EMD, ETFB, EYA4, FHL1, FKTN, FLNA, FOXC1, FOXRED1, FXN, GAA, GATA4, GATA6, GBE1, GDF1, GJA1, GLA, GLB1, GPD1L, HCCS, HCN4, HRAS, JAG1, JPH2, JUP, KCND3, KCNE1, KCNE2, KCNE3, KCNH2, KCNJ2, KCNQ1, KRAS, LAMA2, LAMA4, LAMP2, LDB3, LIAS, LMNA, LRPPRC, LZTR1, MAP2K1, MAP2K2, MED13L, MIB1, MLYCD, MMUT, MTO1, MYBPC3, MYH6, MYH7, MYL2, MYL3, MYLK2, MYO6, MYOZ2, MYPN, NDUFA10, NEXN, NF1, NKX2-5, NOTCH1, NOTCH2, NRAS, NSD1, PCCA, PCCB, PGK1, PIK3CA, PITX2, PKP2, PLN, POLG, PRDM16, PRKAG2, PTPN11, RAF1, RASA1, RASA2, RBM20, RIT1, RRAS, RYR2, SCN1B, SCN3B, SCN5A, SCO2, SDHA, SDHD, SEMA3E, SGCA, SGCB, SGCD, SHOC2, SLC22A5, SLC25A20, SLC25A3, SLC25A4, SNTA1, SOS1, SPRED1, SYNE1, TAFAZZIN, TBX1, TBX5, TCAP, TGFB3, TMEM43, TMEM70, TMPO, TNNC1, TNNI3, TNNT2, TPM1, TSFM, TTN, TTR, VCL, YARS2, ZFPM2*
